# Impact of Preoperative Chemotherapy on Postoperative Renal Dysfunction After Major Abdominal Surgery: A Prospective Observational Study

**DOI:** 10.7759/cureus.64116

**Published:** 2024-07-08

**Authors:** Himanshu S Satapathy, Lalit Sehgal, Manoj Bhardwaj

**Affiliations:** 1 Critical Care Medicine, Tata Main Hospital, Jamshedpur, IND; 2 Liver Transplant Anesthesia/Liver ICU, Manipal Hospital, New Delhi, IND; 3 Anesthesia, Rajiv Gandhi Cancer Institute and Research Center, New Delhi, IND

**Keywords:** postoperative aki, nephrotoxicity, chemotherapy, urine protein to creatinine ratio, cytoreductive surgery, acute kidney injury

## Abstract

Introduction

The administration of anti-cancer drugs and major abdominal surgeries have been independently identified to have a negative effect on renal function. The objectives of the study are to determine the incidence of acute kidney injury (AKI) in patients undergoing major elective abdominal surgery following chemotherapy and identify the independent predictors of postoperative AKI among such cancer patients in a tertiary care cancer institute in North India.

Methods

The prospective observational study included 149 patients aged 18 years or more, scheduled for elective major abdominal cancer surgery. Based on the administration of preoperative chemotherapy, the participants were divided into two study cohorts (Group 1: received preoperative chemotherapy; Group 2: did not receive preoperative chemotherapy). Patients' preoperative characteristics, including the use of preoperative chemotherapeutic agents and intraoperative factors, were evaluated for associations with the development of AKI postoperatively using the Chi-square test and Mann-Whitney U test. Multivariable logistic regression was employed to identify the factors after adjusting for potential confounders.

Results

The overall incidence of postoperative AKI in major abdominal oncosurgery was 24.2% among our study participants, which was significantly higher among patients receiving preoperative chemotherapy (32.4%) as compared to those who did not receive preoperative chemotherapy (16%) (p=0.019). Besides preoperative chemotherapy, the present study also noted that high levels of preoperative urinary protein-to-creatinine ratio (UPCR) and intraoperative use of vasopressors were significantly associated with an increased risk of postoperative AKI development in the final model, after adjustment for all potential confounders. A preoperative UPCR≥0.345 predicted the development of postoperative AKI with 77.8% sensitivity and 83.2% specificity.

Conclusion

Considering the magnitude of the problem, identification of determinants of postoperative AKI in major abdominal surgeries in cancer patients may help anesthetists and surgeons in early detection of AKI, so that prompt precautionary measures can be put in place that can potentially impact prognosis.

## Introduction

Postoperative acute kidney injury (AKI) is a significant risk associated with major abdominal surgeries, associated with significantly worse survival and renal outcomes, yet it has not received the attention it deserves compared to renal dysfunction following cardiovascular surgeries [1]. The risk of AKI is heightened in patients undergoing these extensive surgical procedures. Over the past decade, advancements in cancer research have revolutionized cancer therapy, particularly through the introduction of neoadjuvant chemotherapy for abdominal cancer surgeries [2-4]. While these chemotherapeutic agents, such as cisplatin, ifosphamide, cyclophosphamide, methotrexate, gemcitabine, and various other alkylating agents and immunomodulators, have significantly improved patient outcomes, they also pose a considerable risk to kidney function due to their nephrotoxic effects [5]. Although there has been substantial research on the impact of cytoreductive surgeries followed by perioperative intraperitoneal chemotherapy on postoperative renal functions, the occurrence of renal dysfunction in patients undergoing major abdominal surgery post-chemotherapy remains underexplored. Most existing studies in this domain are either retrospective, focused on a single chemotherapeutic agent, or limited to specific organs [6-8].

Therefore, in the background of limited evidence in this domain, the present study aimed at fulfilling the knowledge gap by prospectively comparing the incidence of AKI in patients undergoing major elective abdominal surgery following chemotherapy to those patients who underwent major elective abdominal surgery without receiving preoperative chemotherapy, in a tertiary care cancer institute in North India. The primary objective of the study was to evaluate the role of preoperative chemotherapy in causing AKI following major elective abdominal surgery and identify the independent predictors of postoperative AKI among such cancer patients in a tertiary care cancer institute in North India. The secondary objective included the identification of a preoperative urine protein-to-creatinine ratio (UPCR) cut-off value to predict the development of postoperative AKI among the study participants as a post-hoc analysis.

## Materials and methods

The present prospective observational study was conducted in a tertiary care hospital in Northern India from September 2017 to December 2018 after approval from the Institutional Review Board (vide reference no. RGCIRC/IRB/77/2017 dated August 16, 2017).

The study included 149 patients aged 18 years or more, scheduled for elective major abdominal cancer surgery. Major abdominal surgery was defined by the criteria of “intra-peritoneal approach under general anesthesia and the predictable length of stay in hospital for patients in a given diagnosis-related group exceeded two days” [9]. The exclusion criteria were as follows: patients with prior history of AKI or chronic kidney disease (based on the availability of previous medical records); received renal replacement therapy (RRT); had pre-existing hydronephrosis; past history of urological surgeries; underwent subsequent surgery within the same hospitalization period/re-exploration. Moreover, during the conduct of cytoreductive surgery, any patient requiring renal or major vessel clamping or underwent ureteric injury or stenting intraoperatively was also excluded.

Based on the administration of preoperative chemotherapy, the participants were divided into two groups; group 1 included patients who underwent major abdominal surgery after receiving chemotherapy, and group 2 included patients who underwent major abdominal surgery without receiving chemotherapy.

Data collection was initiated after obtaining approval from the Institutional Review Board and Institutional Ethics Committee of the concerned hospital. The patients fulfilling the eligibility criteria were explained the purpose and procedure of the study and were ensured on anonymity and confidentiality of the data provided by them. Only those patients who provided written informed consent for participation were included in the study.

In the preoperative period, baseline demographic data, which included age, gender, body weight, and height, was recorded. Body mass index (BMI) was calculated, and patients were classified by the World Health Organization BMI criteria for Asians: underweight (< 18.5 kg/m^2^), normal weight (18.5-22.9 kg/m^2^), overweight (23.0-24.9 kg/m^2^), obesity class I (25.0-29.9 kg/m^2^), and obesity class II (≥ 30.0 kg/m^2^). Thereafter, clinical details (diagnosis, vital parameters, pre-existing comorbidities (hypertension, diabetes, and coronary artery disease) and details of chemotherapy or any other preoperative cancer treatment (if present) and biochemical parameters (hemoglobin (Hb), total leucocyte count (TLC), serum sodium, potassium, albumin, serum creatinine, magnesium, and UPCR) were noted. The estimated glomerular filtration rate (eGFR) was calculated based on the CKD-EPI Creatinine Equation (2021) [10].

During the intraoperative period, the type of surgery conducted, duration of surgery, total intraoperative blood loss, and total volume of intravenous fluids and blood products administered, were recorded for each participant. Perioperatively administered crystalloids included PlasmaLyte (Baxter International Inc., Deerfield, IL) and Sterofundin ISO (B Braun, Melsungen, Germany) and among colloids, Gelofusine (B Braun) was used. Based on previous evidence, intraoperative hypotension was defined by systolic blood pressure (SBP)<90 mm Hg or mean arterial pressure (MAP)<65 mm Hg lasting more than 10 minutes [11]; and use of vasoactive drugs if any, was noted. The first-line vasopressor used in the study was norepinephrine and the second-line drug was vasopressin; doses of vasopressors were titrated to meet the target MAP>65 mm Hg, intraoperatively and postoperatively, irrespective of the cardiac status of the participant. Also, the Hb cut-off for blood transfusion was taken as 8 g/dL according to the institutional management protocol. Intraoperative urine output was monitored hourly through urometer bag collection measurement, and fluid administration was done based on the Pleth variability index (PVI) and at the discretion of the anesthetist, as appropriate.

The patients were followed up for up to 48 hours postoperatively. The primary outcome was the incidence of postoperative AKI. Diagnosis of AKI in the postoperative period was done based on criteria defined by the Kidney Disease Improving Global Outcome (KDIGO) clinical practice guidelines (2012) [12]. Subsequently, KDIGO staging of AKI was also done [12].

The sample size for the study was calculated considering the incidence of AKI as 22.4% from a previous study by Teixeira et al. [13] with a 95% level of confidence, 10% error margin, and allowing for up to 10% loss to follow-up cases. A total of 149 patients were included in the final analysis, with 74 participants in group 1 (who received preoperative chemotherapy) and 75 patients in group 2 (who did not receive preoperative chemotherapy).

Data collected was collated and entered in Microsoft Excel 2016 and statistical analysis was done using the software Statistical Package for Social Sciences (SPSS) version 24.0 (IBM Corp., Armonk, NY). To meet the primary objective, the frequency distribution and association of age group, gender, BMI class, comorbidities, and incidence of postoperative AKI according to the pre-defined study cohorts (Group 1: received preoperative chemotherapy; Group 2: not received preoperative chemotherapy) were represented by cross-tabulation. The association of the incidence of postoperative AKI with preoperative and intra-operative parameters was done through a Chi-square test (for categorical variables) and an independent sample T-test (for continuous variables). The independent factors identified to be significant in bivariate logistic regression were checked for collinearity and analyzed using multivariable regression (stepwise forward method) to identify factors associated with the development of postoperative AKI after adjusting for potential confounders. For the secondary objective, receiver operating characteristic (ROC) curve analysis was undertaken to identify the cut-off value of preoperative UPCR for predicting the development of postoperative AKI. P-value<0.05 was considered as statistically significant.

## Results

Among the 149 consenting patients who participated in the study, the mean age was 54.5±9.5 years, with the majority of the participants in the age group of 45-59 years (55%). The proportion of female patients was 70.5%. An almost equal proportion of participants had a BMI in the normal range (n=71, 47.7%) or were overweight (n=68, 45.6%). Only four patients (2.7%) were found to have class I obesity. The comorbidities included hypertension (n=73, 49%) followed by diabetes mellitus (n=50, 33.6%) and coronary artery disease (n=6, 4%). There were 74 participants in group 1 (who received preoperative chemotherapy) and 75 patients in group 2 (who did not receive preoperative chemotherapy). Except for gender, all the baseline parameters were statistically comparable between the study groups (Table 1).

**Table 1:** Baseline characteristic of study participants according to study groups *p-value was calculated using Chi-square test and a value <0.05 was considered statistically significant BMI: body mass index; HTN: hypertension; DM: diabetes mellitus; CAD: coronary artery disease

Categories	Group 1 (Preoperative chemotherapy)	Group 2 (No preoperative chemotherapy)	Total	P-value*
	n (%)	n (%)	n (%)	
Age				
20-44 years	6 (8.1)	13 (17.3)	19 (12.8)	0.079
45-59 years	47 (63.5)	35 (46.7)	82 (55.0)	
>=60 years	21 (28.4)	27 (36.0)	48 (32.2)	
Gender				
Male	15 (20.3)	29 (38.7)	44 (29.5)	0.014
Female	59 (79.7)	46 (61.3)	105 (70.5)	
BMI class				
Under-nourished (<18.5 kg/m^2^)	1 (1.4)	5 (6.7)	6 (4)	0.435
Normal (18.5-24.9 kg/m^2^)	36 (48.6)	35 (46.7)	71 (47.7)	
Overweight (25-29.9 kg/m^2^)	35 (47.3)	33 (44.0)	68 (45.6)	
Class I obesity (30-34.9 kg/m^2^)	2 (2.7)	2 (2.7)	4 (2.7)	
HTN				
Yes	37 (50.0)	36 (48.0)	73 (49.0)	0.807
No	37 (50.0)	39 (52.0)	76 (51.0)	
DM				
Yes	23 (31.1)	27 (36.0)	50 (33.6)	0.525
No	51 (68.9)	48 (64.0)	99 (66.4)	
CAD				
Yes	3 (4.1)	3 (4.1)	6 (4.0)	1.000
No	71 (95.9)	71 (95.9)	142 (96.0)	
Total	74 (100)	75 (100)	149 (100)	

Among the 74 patients who received preoperative chemotherapy, the combination of paclitaxel and carboplatin (62.2%) was the most predominant chemotherapeutic agent administered, followed by the FOLFOX regime comprising 5-fluorouracil, folinic acid, and oxaliplatin (24.3%). Other less frequently used chemotherapy agents were a combination of gemcitabine, carboplatin, and cisplatin (2.7%), 5-fluorouracil (4.1%), cisplatin (1.4%), capecitabine (4.1%), and imatinib (1.4%).

A total of 36 patients developed AKI in the postoperative period, accounting for the overall incidence of AKI in major abdominal oncosurgery to be 24.2%. The incidence of postoperative AKI among patients receiving preoperative chemotherapy was 32.4%, and it was significantly higher than the incidence of 16% among those who did not receive preoperative chemotherapy (p=0.019) (Table 2).

**Table 2:** Postoperative acute kidney injury according to study groups *p-value was calculated using Chi-square test and a value <0.05 was considered as statistically significant.

Postoperative acute kidney injury	Group 1 (Preoperative chemotherapy)	Group 2 (No preoperative chemotherapy)	Total	P-value*
	n (%)	n (%)	n (%)	
Yes	24 (32.4)	12 (16.0)	36 (24.2)	0.019
No	50 (67.6)	63 (84.0)	113 (75.8)	
Total	74 (100)	75 (100)	149 (100)	

However, the proportion of patients developing different stages of AKI was statistically comparable in both the study groups (p=0.127) (Table 3).

**Table 3:** Stages of AKI among study participants *p-value was calculated using Chi-square test and a value <0.05 was considered as statistically significant AKI: acute kidney injury

Stage of AKI	Group 1 (Preoperative chemotherapy)	Group 2 (No preoperative chemotherapy)	Total	P-value*
	n (%)	n (%)	n (%)	
Stage 1	19 (82.6)	12 (92.3)	31 (86.1)	0.127
Stage 2	4 (17.4)	0	4 (11.1)	
Stage 3	0	1 (7.7)	1 (2.8)	
Total	23 (100)	13 (100)	36 (100)	

**Figure 1:**
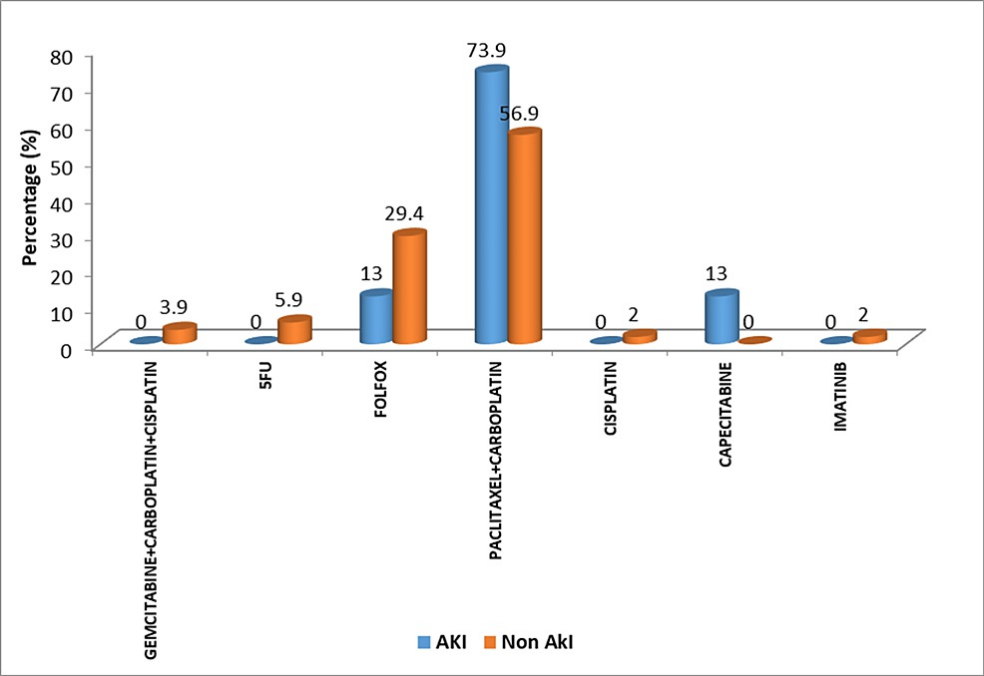
Distribution of study participants according to preoperative chemotherapeutic agent administered and development of postoperative AKI AKI: acute kidney injury

Among patients who received pre-operative chemotherapy, the most frequently administered chemotherapeutic regimens were paclitaxel + carboplatin in 46 patients (62.2%), followed by FOLFOX in 18 patients (24.3%). Other less frequently used regimens were capecitabine (4.1%), 5-fluorouracil (4.1%), gemcitabine + carboplatin + cisplatin (2.7%), cisplatin (1.4%) and imatinib (1.4%). A 37% of the patients who received paclitaxel + carboplatin (17 out of 46), 16.7% of the patients on FOLFOX (three out of 18), and 100% of the patients on capecitabine (three out of three) developed postoperative AKI. The aforementioned regimens constituted 73.9%, 13.05%, and 13.05% of the postoperative AKI cases, respectively (Figure 1).

Also, the two groups were comparable in terms of age, gender, BMI categories and comorbidities. Patients with diagnosed CAD post-PTCA were present equally in both groups, and none of them developed AKI post-surgery. Considering the type of surgery conducted, 75% of the patients developing postoperative AKI underwent gynecological surgeries (radical hysterectomy, total hysterectomy with bilateral salpingo-oophorectomy), while the figure for the same was only 42.5% among those who did not develop AKI; and this difference was statistically significant (p=0.008). While exploring hematological and biochemical parameters assessed preoperatively, it was observed that patients developing postoperative AKI had significantly lower preoperative levels of serum albumin and magnesium, higher value of mean UPCR, but comparable eGFR calculated preoperatively (Table 4). Among the intraoperative parameters assessed, patients developing postoperative AKI had significantly higher amount of blood loss, were administered higher volumes of colloids and blood products and higher proportion of patients received vasopressors. The mean duration of surgery was also higher among patients developing AKI postoperatively (Table 4).

**Table 4:** Association of various preoperative and intraoperative parameters with development of postoperative AKI *p-value was calculated using Chi-square test (for categorical variables) and Mann-Whitney U test (for continuous variables) and a value <0.05 was considered as statistically significant BMI: body mass index; HTN: hypertension; DM: diabetes mellitus; CAD: coronary artery disease; CRS+HIPEC: cytoreductive surgery and hyperthermic intraperitoneal chemotherapy; GI: gastrointestinal; HPB: hepatobiliary; Hb: hemoglobin; SGOT: serum glutamic-oxaloacetic transaminase; SGPT: serum glutamic pyruvic transaminase; ALP: alkaline phosphatase; UPCR: urine protein-to-creatinine ratio; eGFR: estimated glomerular filtration rate; AKI: acute kidney injury

Categories	AKI patients (n=36)	Non-AKI patients (n=113)	Total	P-value*
Age				
20-44 years	6(16.7)	13(11.5)	19(12.8)	0.081
45-59 years	14(38.9)	68(60.2)	82(55)	
>=60 years	16(44.4)	32(28.3)	48(32.2)	
Gender				
Male	4(11.1)	40(35.4)	44(29.5)	0.005
Female	32(88.9)	73(64.6)	105(70.5)	
BMI class				
Underweight (<18.5 kg/m^2^)	0(0)	6(5.3)	6(4)	0.156
Normal (18.5-24.9 kg/m^2^)	15(41.7)	56(49.6)	71(47.7)	
Overweight (25-29.9 kg/m^2^)	21(58.3)	47(41.6)	68(45.6)	
Class I obesity (30-34.9 kg/m^2^)	0(0)	4(3.5)	4(2.7)	
HTN				
Yes	14(38.9)	59(52.2)	73 (49.0)	0.164
No	22(61.1)	54(47.8)	76 (51.0)	
DM				
Yes	12(33.3)	38 (33.6)	50 (33.6)	0.974
No	24(66.7)	75(66.4)	99 (66.4)	
CAD				
Yes	0(0)	6(5.4)	6 (4.0)	0.156
No	36(100)	106(94.6)	142 (96.0)	
Type of surgery				
Gynaecological surgeries	27(75)	48(42.5)	75(50.3)	0.008
Colorectal surgeries	4(11.1)	19(16.8)	23(15.4)	
CRS +HIPEC	3(8.3)	12(10.6)	15(10.1)	
Upper GI surgeries	2(5.6)	18(15.9)	20(13.4)	
HPB surgery	0(0)	16(14.2)	16(10.7)	
Hb (gm/dl)	11.17 (±1.38)	11.53 (±1.66)	--	0.247
Bilirubin (direct) (mg/dl)	0.17 (±0.12) (n=23)	0.14 (±0.10) (n=51)	--	0.171
SGOT (IU/L)	31.50 (±12.56)	35.20 (±40.49)	--	0.593
SGPT (IU/L)	31.97 (±17.07)	37.66 (±79.33)	--	0.678
ALP (IU/L)	107.56 (±34.26)	101.27 (±65.87)	--	0.584
Albumin (g/dl)	3.56 (±0.58)	3.77 (±0.37)	--	0.013
Sodium (mEq/L)	137.22 (3.52)	138.02 (±2.94)	--	0.180
Potassium (mEq/L)	4.43 (±0.49)	4.31 (±0.36)	--	0.123
Magnesium (mg/dl)	1.71 (±0.31)	1.853 (±0.31)	--	0.016
Creatinine (mg/dl)	0.76 (±0.13)	0.95 (±1.53)	--	0.460
UPCR	0.62 (±0.64)	0.24 (±0.13)	--	<0.001
Preoperative eGFR	90.49 (±16.77)	89.88 (±15.64)	--	0.843
Vasopressor				
Yes	26(72.2)	32(28.3)	58(38.9)	<0.001
No	10(27.8)	81(71.7)	91(61.1)	
Crystalloid (mL)	3013.89 (±890.31)	2765.49 (±1420.62)	--	0.325
Colloids (mL)	1486.11 (±731.79)	783.19 (±664.39)	--	<0.001
Mean blood loss(mL)	1101.94 (±1023.74)	599.6 (±608.42)	--	<0.001
Blood products(mL)	551.39 (±587.27)	195.81 (±409.19)	--	<0.001
Duration of surgery (minutes)	343.33 (±64.51)	310 (±78.73)		0.023

Among all these independently identified parameters, preoperative chemotherapy (adjusted odds ratio (AOR), 95% confidence interval (CI): 5.12, 1.23-21.38), high levels of preoperative UPCR (AOR, 95% CI: 3.67, 2.81-18.06) and intraoperative use of vasopressors (AOR, 95% CI: 7.99, 1.72-17.21) retained their significance in the final model, after adjustment for all potential confounders (Table 5).

**Table 5:** Bivariate and multivariable logistic regression to identify factors associated with postoperative AKI *p-value was calculated for binary logistic regression with incidence of postoperative AKI as dependent ** p-value was calculated for multivariable logistic regression (forward-step) model  and a value <0.05 was considered as statistically significant UPCR: urine protein-to-creatinine ratio

Categories	Crude OR (95% CI)	P-value*	Adjusted OR (95% CI)	P-value**
Gender (male)	0.23 (0.08, 0.69)	0.009	0.24 (0.05, 1.32)	0.100
Preoperative chemotherapy (yes)	2.52 (1.15, 5.53)	0.021	5.12 (1.23, 21.38)	0.025
Albumin	0.32 (0.13, 0.79)	0.015	0.26 (0.06, 1.17)	0.078
Magnesium	0.24 (0.07, 0.78)	0.018	0.27 (0.04, 2.09)	0.209
UPCR	4.09 (1.05, 10.78)	<0.001	3.67 (2.81, 18.06)	<0.001
Vasopressor (yes)	3.79 (1.54, 9.37)	0.004	7.99 (1.72, 17.21)	0.008
Blood loss	1.01 (1.00, 1.02)	0.001	0.99 (0.98, 1.01)	0.399
Duration of surgery	1.06 (1.01, 1.11)	0.025	1.01 (0.99, 1.02)	0.638

**Figure 2:**
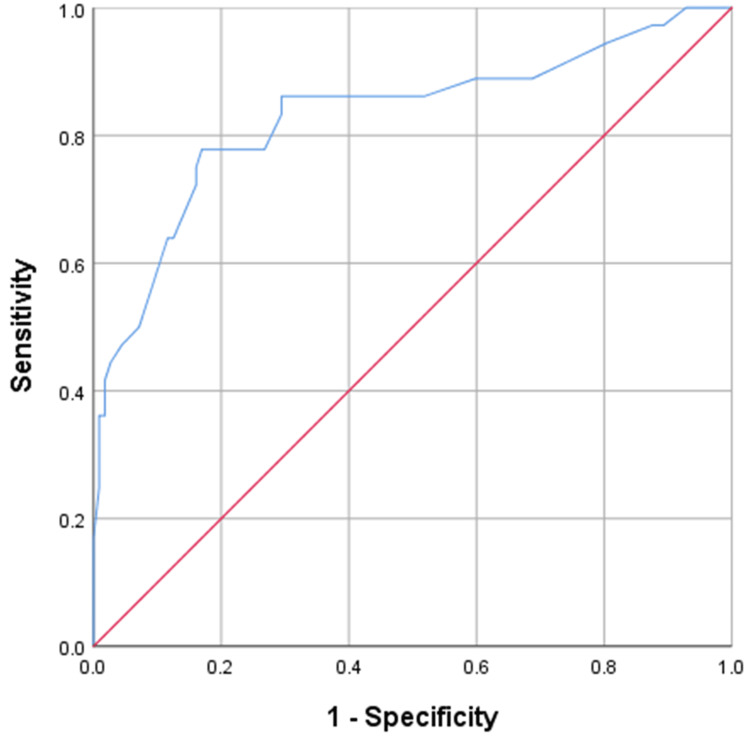
ROC curve for preoperative urine protein-to-creatinine ratio (UPCR) predicting development of postoperative AKI ROC: receiver operating characteristic; AKI: acute kidney injury

Upon conducting ROC analysis of preoperative UPCR values in predicting postoperative AKI, the area under curve was 0.831, and a cut-off value of UPCR ≥ 0.345 identified to yield 77.8% sensitivity, 83.2% specificity, 59.6% positive predictive value, 92.2% negative predictive value and 81.9% diagnostic efficacy (Figure 2).

## Discussion

Postoperative AKI is multifactorial and has a complex pathogenesis. There is a dearth of literature on determinants of AKI following major abdominal surgery specifically in cancer patients. In the present study, the overall incidence of postoperative AKI was 24.2%; however, the incidence was 32.4% among patients who received additional preoperative chemotherapy, which was considerably higher than 16% among those who merely underwent cytoreductive abdominal surgery without receiving prior chemotherapy. The incidence was found to be slightly higher than the study conducted by Teixeria et al. where the incidence was found to be 22.4% [13]. They included all the abdominal surgery while only cancer surgeries were included in the present study. Vaught et al. [14] observed that the overall incidence of AKI was 13%. While AKI, in their study, was defined by the RIFLE criteria included patients undergoing gynecological surgeries only; our study participants comprised patients undergoing various types of major abdominal cancer surgeries and also patients who had received chemotherapy prior to surgery, which may account for the higher incidence observed.

The kidneys play a crucial role in the elimination of chemotherapeutic agents and their metabolites, which are most commonly nephrotoxic, making the patient susceptible to multiple renal complications, including interstitial nephritis and acute tubular damage [4]. Concurrently, the development of AKI following major abdominal surgery may be facilitated by one or a combination of preoperative risk factors, intraoperative and postoperative events; mediated by injury pathways affecting kidney microcirculation, inflammation, and oxygen demand [14]. The present study observed that the chemotherapeutic regimen of paclitaxel + carboplatin contributed to the majority of the postoperative AKI cases, followed by the FOLFOX regimen and capecitabine. Although a platinum-based drug, cisplatin is notorious for its high nephrotoxic potential, the same could not be established in the present study because very few patients were administered cisplatin alone or in combination with other chemotherapeutic agents prior to abdominal surgery. While the use of nephrotoxic anti-cancer drugs is one of the major preoperative risk factors, other unavoidable processes causing fluid depletion throughout surgery initiate right from the preoperative period as a result of routine nil-per-oral regimens and fluid loss from the organ pathology itself, through intraoperative events such as blood loss, extravasation of fluids to the third space and insensible losses. In the present study, PVI-guided fluid therapy was followed, and the adequacy of fluid status was assessed through the maintenance of a target MAP>65mm Hg. Utmost precautions were taken to avoid fluid imbalances perioperatively. In patients undergoing cytoreductive surgery and hyperthermic intraperitoneal chemotherapy (CRS+HIPEC), systemic hyperthermia was prevented by active cooling with cold packs and infusion of cold saline (at 4-degree Celsius). As documented in the literature, a further cause for increased fluid loss under general anesthesia is mechanical ventilation of the intubated patient [15]. However, in this study, all patients were extubated in the immediate postoperative period.

After a cascade of renal responses to hypoperfusion, ischemia to the renal medulla occurs; which along with the impact of anesthetic agents and surgical stress leads to increased plasma renin activity and secretion of antidiuretic hormones, and a compensatory adjustment in the overall fluid-electrolyte homeostasis. Moreover, non-hemodynamic factors such as activation of pro-inflammatory response during abdominal surgery, coupled with further tubular injury in the post-ischemic phase caused by reactive oxygen species and tissue inflammation leads to a rapid decline in kidney function; thereby resulting in acute onset of kidney injury postoperatively [16]. Our observation provides ground for speculation that the combined impact of one or more cycles of chemotherapy preoperatively followed by major cytoreductive abdominal surgery may predispose the kidneys to recover inadequately following repeated insults to the already compromised bodily functions attributed to the carcinogenic status of the patient.

In the quest for the determinants of postoperative AKI following major abdominal cytoreductive surgeries, we found females were three times more prone to develop AKI than males (30.48% vs 9.09%) (P=0.005). In contrast to the finding, Rewa et al. [17] and Liangos et al. [18] showed that male gender was a risk factor for AKI development postoperatively. The difference may be explained by the higher number of female patients in the present study.

The present study also identified hypoalbuminemia and higher urine protein to creatinine ratio as independent risk factors for the development of postoperative AKI. Our observations were supported by Sin et al. [19] who also informed preoperative hypoalbuminemia is a significant risk factor for postoperative AKI. Marouli et al. [20] reported that an abnormal preoperative urine albumin to creatinine ratio (UACR) at a magnitude >30 mg/gm had a five-fold higher risk of developing AKI irrespective of preoperative renal functions or other comorbidities. To the best of our knowledge, our study was second to Marouli et al. [20] to report a strong association between preoperative UPCR and postoperative AKI, to report it in major abdominal surgeries, a component that prior predictive models have failed to consider. While serum creatinine has been considered a less sensitive and a lagging indicator of AKI, evidence from the literature suggests that UPCR and UACR are reliable estimates of daily excretion values of creatinine [21]. However, it must be kept in mind that our study participants were oncology patients, and many of the patients who developed postoperative AKI had received preoperative chemotherapy, and both these conditions might have already set the ground for kidney injury even prior to conducting major cytoreductive abdominal surgery. Despite these circumstances, the exhibition of a strong and consistent association between preoperative UPCR and the development of postoperative AKI, even after adjusting for potential confounders in the final regression model, is suggestive of UPCR as a potential biomarker in predicting postoperative AKI which requires further exploration.

In line with the pathophysiological explanation for intraoperative fluid depletion in major abdominal surgeries, we noted a significantly higher volume of blood loss, which corroborated with higher volume replacements through colloids and blood products, in patients who later developed postoperative AKI, but no substantial difference in the volume of crystalloids infused. Similar observations were reported by Teixeira et al. [13] and Marouli et al. [20] Besides, the postoperative AKI group had a significantly higher mean duration of surgery than the non-AKI group. Our observation could be explained by the fact that a longer duration of surgery was directly related to a longer duration of anesthesia, as also reported by Teixeira et al. [13]. Nevertheless, the aforementioned variables lost their statistical significance when adjusted with each other in the multivariable regression model.

Despite the availability of established literature on the nephrotoxic potential of chemotherapeutic agents and the renal impact of abdominal surgeries independently, there is no literature available that explored the combined renal impact of various pre-operative chemotherapy regimens followed by abdominal cytoreductive surgeries, especially on the Indian population with solid malignancies. Moreover, the present study went a step ahead to speculate on the role of UPCR as a potential biomarker in predicting postoperative AKI.

The major limitation of our study was the use of creatinine as a conventional marker of AKI, which does not adequately and promptly reflect changes in glomerular filtration rate during a dynamic state and can even rise in the early stages of AKI, when GFR is declining, due to tubular secretion of creatinine. The most significant drawback of creatinine is that it is not a real-time biomarker; resulting in a creatinine blind range as levels may not increase until renal function is impaired. Diagnosis by serum creatinine for AKI may even be delayed by one to three days after the initial injury to the kidney has occurred [22,23]. Secondly, the patients did not undergo diethylenetriaminepentacetate (DTPA) scans, and therefore any cases of sub-clinical AKI could not be identified. Also, a detailed history of chronic comorbidities, especially long-standing diabetes or insulin-dependent diabetes mellitus, which increases the risk of sub-clinical CKS and perioperative AKI, was not taken into account. Thirdly, although the incidence of postoperative AKI has been reported for specific chemotherapeutic regimens, chemotherapy-related parameters like specific dose, duration, and interval between chemotherapy and surgery were not taken into consideration in the present study. Moreover, the present study comprised cancer patients undergoing major abdominal surgeries involving various organ systems. Therefore, non-uniformity in the type of surgical procedures is a limitation, as the extent of surgical dissection, their physiological impact, and the duration of surgery may vary. Other markers, such as neutrophil gelatinase-associated lipocalin (NGAL) may be considered in future studies to identify early AKI.

## Conclusions

In our study, the incidence of postoperative AKI in patients receiving preoperative chemotherapy was 32.4%. The majority of the patients developed stage 1 AKI which indicates that kidney injury in the immediate postoperative period is still reversible if managed promptly. Besides preoperative chemotherapy, high levels of preoperative UPCR and intraoperative use of vasopressors were significantly associated with increased risk of postoperative AKI development. Considering the magnitude of the problem, identification of determinants of postoperative AKI in major abdominal surgeries in cancer patients may help anesthesiologists and surgeons in the early detection of AKI, so that prompt precautionary measures, such as optimizing fluid therapy and vasopressors, are put in place that can potentially impact prognosis.
